# Trade-offs from a family perspective: considerations in choosing between omalizumab and complementary alternative medicine for pediatric severe asthma

**DOI:** 10.3389/fphar.2025.1621101

**Published:** 2025-07-31

**Authors:** Hang Chen, Yan Xu, Jian-Li Qiu, Zhi-Wei Guan, Mu-Mu Wei, Yi Zhang, Ji-Xiang Xu, Hong-Tao Cui

**Affiliations:** ^1^ School of Pediatrics, Henan University of Chinese Medicine, Zhengzhou, Henan, China; ^2^ Department of Pediatrics, The First Affiliated Hospital, Henan University of Chinese Medicine, Zhengzhou, Henan, China; ^3^ Chongqing Hospital of Traditional Chinese Medicine, Chongqing, China

**Keywords:** pediatric severe asthma, family perspective, omalizumab, complementary alternative medicine (CAM), treatment decision-making, risk perception, treatment burden, shared decision-making (SDM)

## Abstract

The management of pediatric severe asthma poses significant challenges for families. When faced with the choice between targeted biologics like omalizumab and widely used complementary alternative medicine (CAM), families navigate a complex decision-making process influenced by multiple factors. This review adopts a family-centered perspective to systematically analyze key factors influencing this trade-off: treatment goals (extending beyond clinical metrics to focus on quality of life), risk perception (shaped by subjective constructs and lacking direct evidence for comparative risk assessments), treatment burden (often overlooked hidden costs), and the current state of shared decision-making (SDM). Analysis reveals that family decision-making is a multidimensional construct shaped by four core elements: value systems, lived experiences, risk perception patterns, and tolerance for treatment burden. Notably, the significant gap in risk perception evidence leads to subjective risk assessments dominating decisions, particularly in CAM choices. Treatment burden, a critical hidden cost, is often marginalized in decisions, hindering effective SDM. Health equity further profoundly impacts choices. The conclusion emphasizes the need for clinical practice to shift toward family-centered care by addressing real-world needs, routinely evaluating treatment burden, optimizing risk communication, overcoming SDM barriers, and promoting health equity. Future research must fill evidence gaps in risk perception, develop SDM tools, and address culturally diverse family needs.

## 1 Introduction

Pediatric asthma, the most prevalent chronic respiratory disease in children globally, profoundly impacts patients’ and families’ quality of life (QoL). Severe pediatric asthma, with a prevalence of 6.9%, is less common but poses unique management challenges (Haktanir Abul and Phipatanakul, 2019). Clinically, it is defined as asthma that remains uncontrolled despite high-intensity therapy (e.g., GINA Step 4 or 5 treatments, including high-dose inhaled corticosteroids [ICS] plus long-acting β2-agonists [LABA]) or requires chronic oral corticosteroids (OCS) (Grunwell and Fitzpatrick, 2025; Bush, 2024). These children experience frequent exacerbations and require intensive interventions (Larenas-Linnemann et al., 2025).

Advances in precision medicine have introduced new options like omalizumab, a biologic targeting IgE-mediated pathways, offering hope for specific phenotypes (Di Cicco et al., 2025). Biologic use typically follows inadequate response to conventional therapy, with SDM emphasizing efficacy (limited pediatric data), family goals, administration (e.g., subcutaneous injections), burden, and safety uncertainties (e.g., anaphylaxis risk) (Anderson et al., 2023; Cornelius et al., 2024).

Meanwhile, CAM—encompassing herbal remedies, acupuncture, homeopathy, and dietary interventions—is widely used globally (Heidrich et al., 2017). Families choose CAM due to concerns about conventional drug side effects (especially steroids), preferences for “natural” or “holistic” approaches, cultural traditions, or desires to reduce medication dependence (Heidrich et al., 2017; Berg et al., 2016).

Omalizumab and CAM represent divergent paradigms in evidence base, risk profiles, burden, and cultural acceptance. While omalizumab has robust efficacy data, it carries high costs (Courtney et al., 2005) and safety concerns (Anderson et al., 2023). CAM, though perceived as “gentler,” lacks standardization, with variable efficacy/safety evidence (Heidrich et al., 2017). Alarmingly, CAM use may correlate with reduced adherence to conventional therapy and worsened control (Adams et al., 2007), while the “natural = safe” myth underestimates risks like herb-drug interactions (Heidrich et al., 2017).

Families thus face complex trade-offs, balancing efficacy, risks, burden, and values. Despite clinical research focusing on objective outcomes, understanding family decision-making is vital for patient-centered care. However, systematic explorations of family perspectives—particularly risk perception and burden—remain scarce.

This review synthesizes literature to address: (1) Family treatment goals and the role of PROs; (2) Risk perception construction for omalizumab vs. CAM; (3) Unique treatment burdens (including psychosocial impacts); (4) Family roles in SDM and current barriers.

Through an in-depth analysis of these questions, this review expects to shed light on the fundamental factors influencing families’ choices regarding treatment, identify communication barriers and unmet needs in current clinical practice, and consequently provide an evidence base and theoretical foundation for optimizing management strategies for severe childhood asthma, facilitating effective shared decision-making between clinicians and patients, and ultimately enhancing the health outcomes and overall wellbeing of children with the condition and their families.

## 2 Methods

### 2.1 Review type and purpose

This study employs a Narrative Review methodology to systematically synthesize existing literature, aiming to comprehensively understand the perspectives, experiences, decision-making considerations, risk perceptions, treatment burdens, and related physician-patient communication and health equity issues among families of children with severe asthma (including parents, caregivers, and patients themselves) when choosing between omalizumab and Complementary and Alternative Medicine (CAM).

### 2.2 Literature search strategy

To ensure comprehensiveness and relevance, this study primarily searched the PubMed/MEDLINE database, supplemented by additional literature searches to validate key arguments, fill evidence gaps, and enhance analytical depth. The search strategy integrated four core concept groups using Medical Subject Headings (MeSH Terms) and free-text terms connected by Boolean operators (AND, OR): (1) Disease concepts: “Asthma”, “pediatric asthma”, “severe asthma”, “childhood asthma”, “difficult asthma” (2) Population concepts: “Child”, “Adolescent”, “parent”, “family”, “caregiver” (3) Treatment concepts: “Omalizumab”, “omalizumab”, “Anti-IgE”, “Biological Therapy”, “biologics”, “monoclonal antibody”, “Complementary Therapies”, “complementary alternative medicine”, “CAM”, “Medicine, Traditional”, “traditional medicine”, “Herbal Medicine”, “herbal medicine”, “Acupuncture Therapy”, “acupuncture”, etc. (4) Core themes and outcomes: “Patient Preference”, “perspective”, “view”, “attitude*”, “Beliefs”, “treatment beliefs”, “expectation”, “goals”, “priorities”, “Patient Reported Outcome Measures”, “patient reported outcomes”, “Quality of Life”, “Risk Perception”, “risk perception”, “safety concerns”, “fear”, “uncertainty”, “Cost of Illness”, “treatment burden”, “burden of treatment”, “lived experience”, “daily challenges”, “Psychosocial Impact”, “Decision Making”, “decision making process”, “treatment choice factors”, “Shared Decision Making”, “shared decision making”, “Communication Barriers”, “communication barriers”, “Patient Compliance”, “adherence barriers”, “Trust”, “Physician-Patient Relations”, “Healthcare Disparities”, “health equity”, “disparities”, “Socioeconomic Factors”, “cultural factors”.

The language is restricted to English. The timeframe is focused on the last 15 years, with inclusion of seminal early studies and the latest publications to ensure breadth and timeliness.

### 2.3 Inclusion and exclusion criteria

Literature screening adhered to the following criteria: Inclusion criteria specified research papers published in peer-reviewed journals (including quantitative, qualitative, or mixed-methods studies), systematic reviews, meta-analyses, clinical guidelines, or expert consensus; studies involving children (age < 18 years) diagnosed with severe or refractory asthma; and studies exploring aspects related to family (parents, caregivers, or the child themselves) perspectives, experiences, decision-making processes, risk perceptions, treatment burden, quality of life, physician-patient communication, or health equity concerning omalizumab or Complementary and Alternative Medicine (CAM). Conversely, exclusion criteria included: studies focusing solely on adult asthma; studies focusing solely on biologics other than omalizumab or conventional therapies other than CAM; studies focusing exclusively on clinical efficacy, pharmacokinetics, or purely biomedical mechanisms without addressing family perspectives, experiences, or decision-making; and non-full research articles such as conference abstracts, letters, or editorial comments.

### 2.4 Literature screening and data synthesis

The literature screening process involved the following steps: First, an initial screening was performed by reviewing titles and abstracts to exclude clearly irrelevant records. Subsequently, the full texts of potentially relevant articles were retrieved and assessed in detail to determine final eligibility based on the inclusion criteria. For studies meeting the criteria, key information was extracted—including study design, study population, treatment methods used, and core findings related to the central themes of this review (e.g., expectations and goals, risk perception, treatment burden, decision-making processes and shared decision-making, equity). This information was then summarized and organized. Given the narrative nature of this review, a formal risk of bias assessment was not conducted for the included literature; however, priority was given during selection to studies with higher-quality research designs and richer informational content. Data synthesis focused on integrating findings from the different studies, identifying common themes and divergent perspectives, with the aim of constructing a comprehensive picture of family perspectives in the treatment of severe childhood asthma.

## 3 Results

Based on the literature search results, this section elaborates in-depth on the core considerations for families facing choices between omalizumab and CAM in the treatment of severe childhood asthma. The analysis is structured around the following four dimensions, aiming to enhance academic rigor and evidence support.

### 3.1 Treatment expectations and goals: focusing on quality of life and patient-reported outcomes

Literature analysis reveals that family expectations and goals for severe childhood asthma treatment are multidimensional and individualized, significantly extending beyond traditional clinical indicators (such as improved lung function or reduced frequency of acute exacerbations). Improving and maintaining a good quality of life (QoL) is one of the most central concerns for families (Gandhi et al., 2013; Zhou et al., 2025; Hossny et al., 2017; Aldirawi et al., 2025; Kasse et al., 2024). Families expect treatment to enable the child to participate maximally in normal daily activities, including unrestricted school attendance, participation in sports, social interactions, and enjoyment of leisure time (Gandhi et al., 2013; Zhou et al., 2025). Furthermore, whether the treatment can reduce the disease’s interference with overall family functioning (such as parental work or family leisure activities) is also an important consideration for families (Khaleva et al., 2025).

Patient-Reported Outcomes (PROs), as key indicators for measuring treatment effectiveness and reflecting the patient’s actual experience, are increasingly recognized for their importance (Zhou et al., 2025; Hossny et al., 2017; Williams et al., 2023). PROs tools specifically for childhood asthma, such as the Pediatric Asthma Quality of Life Questionnaire (PAQLQ), the Asthma Control Questionnaire (ACQ), and the recently developed electronic Patient-Reported Asthma Symptom Diary (ePASD) (Majellano et al., 2023), aim to capture symptoms, activity limitations, and psychological states from the perspective of the child and family. Research confirms that the level of asthma control is closely related to children’s QoL (Zhou et al., 2025; Kasse et al., 2024; Khaleva et al., 2025). Therefore, treatment decisions should place the improvement of QoL and PROs at a core position, equally important as clinical indicators. Identifying and prioritizing research focuses of concern to patients and caregivers (such as strategies for improving QoL) is also crucial for guiding future research directions (Ip et al., 2018).

Beyond QoL, families generally expect treatment regimens to minimize side effects, particularly concerning the long-term use of controller medications (like ICS) or potent drugs (like OCS) (Heidrich et al., 2017). The unknown long-term safety of biologics such as omalizumab also constitutes a significant concern for some families (Anderson et al., 2023). This concern about medication safety, especially “steroid phobia” (Michalopoulou et al., 2016), becomes a significant driver for seeking CAM, with many families hoping CAM can help reduce dependence on conventional medications (Heidrich et al., 2017). Literature confirms that fear of long-term medication use is one of the predictors of CAM use (Berg et al., 2016).

Although asthma is medically considered incurable, some families, especially after experiencing poor efficacy with multiple conventional therapies, may still hold expectations of finding a “cure” or “root cause” solution, sometimes leading them to explore CAM (Berg et al., 2016). Furthermore, during the treatment process, families seek not only relief from physiological symptoms but also emotional and psychological support. They expect understanding, empathy, and effective communication from healthcare professionals (Doerr, 2001), and desire a degree of autonomy and control in managing their child’s health. For some CAM users, the longer consultation times, explanatory frameworks more aligned with their cultural beliefs, or different practitioner-patient relationship models offered by CAM may fulfill needs not fully met within the conventional healthcare system (Heidrich et al., 2017; Van Sickle et al., 2003).

Expectations specific to therapies: Choosing omalizumab is typically an “escalation” choice after conventional high-dose therapies have failed, with the core expectation being a significant reduction in acute exacerbations, decreased OCS dependence, and improved QoL (Di Cicco et al., 2025; Anderson et al., 2023). Conversely, expectations for seeking CAM tend to be more diverse; beyond general hopes, they may include beliefs in its “natural and gentle” properties, its ability to “strengthen the constitution,” influences from cultural beliefs, or the search for alternative explanatory frameworks for the disease (Heidrich et al., 2017; Pongdee and Li, 2025).

In summary, family expectations for the treatment of severe childhood asthma are multidimensional, with QoL and PROs serving as core benchmarks. Concerns about side effects and long-term medication dependence, the hope for a “cure,” and the need for emotional support and autonomy collectively shape families’ treatment goals and profoundly influence their choices between different therapies like omalizumab and CAM.

### 3.2 Risk perception and safety narratives: subjective construction and evidence gaps

Families’ perception of potential risks associated with different treatment options and their beliefs about safety are key psychological factors influencing their treatment choices and adherence. However, empirical research directly and deeply exploring families’ specific risk perceptions regarding omalizumab and various types of CAM is extremely scarce in the existing literature, constituting a significant evidence gap. Therefore, the current understanding of this issue is largely based on inferences drawn from related studies (such as motivations for CAM use, reasons for treatment non-adherence, etc.) and the application of risk perception theories.

#### 3.2.1 Subjective construction of omalizumab-related risks

The process by which families subjectively construct omalizumab-related risks profoundly influences their decisions, but literature directly studying families’ specific perceptions remains scarce. Based on the drug’s characteristics and principles of risk communication, it can be inferred that family concerns about risk primarily focus on several aspects. First, regarding known but rare serious risks, such as anaphylaxis, although clinical data show an incidence of only 0.1%–0.2% (Zhang et al., 2023), its potential lethality may trigger significant risk amplification phenomena. This might manifest as parents potentially overestimating its severity and actively searching for adverse reaction cases to confirm their worries. Negative emotions triggered by the injection setting (e.g., fear of needles) might further intensify this risk perception (Bacharier and Jackson, 2023). Second, due to relatively limited long-term medication data in the pediatric population (Zhang et al., 2023), families hold persistent concerns about potential unknown long-term risks, especially regarding possible effects on immune system development in children younger than 6 years, and long-term malignancy risk (although current evidence shows no clear association) (Zhang et al., 2023). This state of “unknown unknowns” often leads some families to hesitate or opt for delayed treatment. Furthermore, beyond the drug itself, the treatment process entails a series of secondary risks, including procedural risks (such as injection site reactions with an incidence of 11%–18% (Ma et al., 2023), exposure risks for severely allergic children during clinic visits), management risks (e.g., drug ineffectiveness due to cold chain disruption, treatment interruption caused by appointment system issues), and potential psychological risks from repeated injections (e.g., treatment fatigue). Ultimately, families’ understanding and acceptance of risks largely depend on the source and presentation of information, as well as their trust in the information provider (primarily the physician). The completeness of physician communication (e.g., whether rare adverse reactions are proactively mentioned), the authority of the information source (e.g., drug labels versus social media discussions), and the format of risk presentation (e.g., differences between absolute and relative risk) all significantly influence the intensity of family risk perception.

#### 3.2.2 Cognition of CAM-Related risks and belief biases

Compared to omalizumab, families’ risk perception regarding Complementary and Alternative Medicine (CAM) exhibits different characteristics and is often accompanied by significant cognitive biases. A prevalent belief that “natural equals safe,” i.e., the assumption that therapies derived from nature (like herbal remedies) are inherently safer than synthetic drugs (Heidrich et al., 2017), can lead families to systematically underestimate the potential risks of CAM. For example, although studies indicate that herbal preparations can cause liver injury [e.g., accounting for 25% of drug-induced liver injury (Handelman et al., 2004)], many families may still insist that “natural ingredients are absolutely safe” and rarely proactively seek information on CAM product side effects. This stems from a cognitive bias that incorrectly equates “natural” with “harmless.” Concurrently, families may have insufficient awareness of many inherent risks of CAM, including potential interactions between herbs and conventional medications (e.g., synergistic toxicity between ephedrine and beta-agonists), uncontrollable dosages due to unknown or unstable active ingredients, the possibility of product contamination or adulteration, and the risk of delaying more effective conventional treatment due to reliance on unproven CAM therapies. Information sources for CAM are extremely diverse and often lack rigorous scientific validation and official oversight, making it difficult for families to obtain accurate and reliable risk information. Furthermore, significant regulatory discrepancies exist between CAM products and conventional drugs regarding pre-market safety proof, adverse reaction monitoring, and quality control standards, further exacerbating information asymmetry. Some families, fearing physician disapproval or lack of understanding, choose not to disclose their CAM use to doctors. This not only hinders open communication but also increases the risk of drug interactions and adverse events. Research also suggests that positive beliefs about CAM are sometimes associated with reduced adherence to conventional therapy and worsening asthma control (Adams et al., 2007; Gandhi et al., 2013).

#### 3.2.3 The role of explanatory models (EMs) and treatment beliefs

A deeper understanding requires introducing the concept of Explanatory Models (EMs). A study focusing on families of inner-city children with asthma found that parents held various concerns about medications (such as unknown side effects, addiction, too much medication, etc.). These beliefs, based on their own EMs, directly led to non-adherent behaviors like reducing or discontinuing medication (Ip et al., 2018; Ischander and Lozowski-Sullivan, 2022). Similarly, “steroid phobia” is also a common negative treatment belief that can affect adherence and treatment choices (Zhao et al., 2023; Bingemann et al., 2024). Families’ specific beliefs about the disease (e.g., believing asthma is caused by “catching a cold” or “weak constitution”) and treatment (e.g., believing CAM can “treat the root cause” while Western medicine only “treats the symptoms”) profoundly influence their risk assessment and treatment choices.

In summary, in the treatment of severe childhood asthma, family risk perception is a subjective construction process, deeply influenced by their explanatory models, treatment beliefs, and tolerance for uncertainty. Existing evidence suggests that risk perception regarding omalizumab may be amplified due to its technical nature, known serious consequences, and unknown long-term effects; whereas risks associated with CAM may be underestimated due to “natural bias” and insufficient information. Bridging the gap between objective risk data and families’ subjective risk perception is a core challenge for effective risk communication and promoting informed decision-making. This field urgently requires high-quality empirical research to fill the evidence gaps.

### 3.3 Treatment burden: daily challenges and psychosocial impacts beyond the clinic

Severe asthma itself and its management process impose a heavy multidimensional burden on children and their families, significantly impacting their quality of life (QoL) and psychosocial functioning (Jones et al., 2018; Ohtsuka et al., 2005; Majellano et al., 2023; Golding et al., 2021). The concept of Treatment Burden emphasizes that, in addition to medication side effects, the entire “workload” involved in executing the treatment plan, and the negative impact of this work on family daily life and psychological state, are crucial components constituting the burden (Graves et al., 2007). The literature search provided further evidence on this topic.

#### 3.3.1 The combined burden of disease and treatment

The inherent burden of severe asthma is already substantial, including activity limitations due to symptoms, sleep disturbances caused by nocturnal awakenings, potential impacts on academic performance, and the resulting persistent psychological stress on both the child and caregivers (especially mothers who often bear the primary caregiving responsibility) (Jones et al., 2018; Ohtsuka et al., 2005; Ischander and Lozowski-Sullivan, 2022; Majellano et al., 2023; Golding et al., 2021; Valero-Moreno et al., 2018), such as anxiety and depressive symptoms. These burdens constitute the baseline challenges of families’ daily lives.

Building upon this foundation, the treatment process itself imposes additional, non-negligible burdens. First, the cost in terms of time and energy is extremely high (Jones et al., 2018). Families need to invest substantial time in daily medication management (e.g., using inhaled medications, cleaning nebulizer equipment), closely monitoring symptom fluctuations, being constantly prepared to manage acute exacerbations, and frequently traveling to healthcare facilities for follow-up visits or specific treatments (for instance, omalizumab requires regular subcutaneous injections). Additionally, tasks such as communicating the child’s condition to school teachers and coordinating special needs during school hours further consume family time and energy. Notably, for families choosing Complementary and Alternative Medicine (CAM), burdens also exist, such as preparing herbal remedies themselves, following special dietary management plans, or finding and regularly visiting specific CAM practitioners, all of which require significant investment of time and effort.

Second, economic pressure is another major challenge faced by families with severe asthma. Beyond direct medical costs (like consultation and examination fees) and medication expenses (especially for biologics like omalizumab, which are often very costly) (Courtney et al., 2005; Adams et al., 2007), there are also numerous often-overlooked indirect costs, such as transportation expenses to and from the hospital, and loss of parental work time due to accompanying the child to appointments or providing care. For CAM therapies, since many are not covered by health insurance, their costs can also impose an additional financial burden on families.

Finally, the psychosocial impact is particularly profound. The chronic stress from ongoing disease management, concerns about treatment outcome uncertainty, fear of potential side effects from medications (especially steroids or biologics), and the disruption caused by the illness to overall family functioning (potentially affecting marital relationships, reducing attention to other healthy children, limiting family leisure and social activities, etc.) all exert a continuous negative impact on the mental health of family members (Jones et al., 2018; Ohtsuka et al., 2005; Ischander and Lozowski-Sullivan, 2022; Majellano et al., 2023; Golding et al., 2021; Valero-Moreno et al., 2018). Existing research has confirmed that families of children with chronic lung diseases (including severe asthma) commonly experience significant psychosocial stress and caregiver burnout (Bingemann et al., 2024; Adams et al., 2004). Similar caregiver burden and psychological stress have also been observed in families of children with food allergies, a common comorbidity with asthma (Golding et al., 2021; Graves et al., 2007; Ziaian et al., 2006; Khalsi et al., 2024). This complex burden, formed by the superposition of the disease and its treatment, constitutes the core challenge in the daily lives of families with severe childhood asthma.

#### 3.3.2 Impact of treatment burden on decision-making and adherence

Treatment burden theory emphasizes that negative consequences arise when the treatment “workload” exceeds the family’s “capacity” to cope (Valero-Moreno et al., 2018; Graves et al., 2007). Research confirms that families subconsciously or consciously weigh the burden of disease against the burden of treatment (Amirav et al., 2020). When the treatment burden feels overwhelming (e.g., if the regimen is too complex or interferes with daily life), families may adopt strategies of “volitional nonadherence,” such as independently reducing medication dosage or frequency, seeking a balance between an acceptable level of control and a tolerable treatment burden (Valero-Moreno et al., 2018; Adams et al., 2004; Ziaian et al., 2006; Graves et al., 2007; Ramsey et al., 2023; Amirav et al., 2020). Therefore, failure to adequately identify and address the treatment burden perceived by families is a significant contributor to poor treatment adherence and ultimately suboptimal clinical outcomes. Treatment burden has been shown to significantly impact the QoL of children with asthma, similar to findings in other chronic conditions such as diabetes and cystic fibrosis (Khalsi et al., 2024; Prather et al., 2020).

In summary, managing severe childhood asthma imposes heavy burdens on families, encompassing time, energy, financial, and psychosocial aspects. While omalizumab and CAM therapies attempt to alleviate disease symptoms, they may also introduce their own unique treatment burdens. This treatment burden is a critical factor influencing family QoL, treatment decisions, and adherence, yet it is often overlooked in clinical practice. Systematic assessment and management of treatment burden should be an integral component of family-centered care models.

### 3.4 Decision-making processes and shared decision-making (SDM): mechanisms, barriers, and facilitators

Treatment decisions for severe childhood asthma are not simple technical choices but rather complex interactive processes, deeply influenced by family characteristics, the quality of the physician-patient relationship, and the healthcare system environment. Shared Decision-Making (SDM) is widely recognized as the ideal model for achieving patient-centered care. It emphasizes the joint participation of both healthcare providers and patients (families) in the decision-making process, based on the best available clinical evidence combined with the patient’s (family’s) values and preferences. This review provides richer evidence for understanding the practice, barriers, and facilitators of SDM in the management of childhood asthma.

#### 3.4.1 Key factors influencing family decision-making

In addition to the previously mentioned factors such as disease perception, health literacy (Amirav et al., 2020; Ramsey et al., 2023), information sources and quality, past experiences and expectations, and socioeconomic and cultural backgrounds (Pongdee and Li, 2025; Prather et al., 2020; Santos Malavé et al., 2019; Reeves et al., 2020; Bacharier and Jackson, 2023; Rivera-Spoljaric et al., 2014; Gijsen et al., 2024; Sleath et al., 2011), families’ treatment beliefs (Ip et al., 2018; Ischander and Lozowski-Sullivan, 2022) and their weighing of the pros and cons of different treatment options are crucial. Choosing omalizumab involves a comprehensive consideration of efficacy, burden, and risks, representing a typical scenario requiring SDM (Anderson et al., 2023; Cornelius et al., 2024). The choice of CAM, on the other hand, may be driven by factors such as fear of conventional medications (Courtney et al., 2005), cultural preferences (Pongdee and Li, 2025; Bacharier and Jackson, 2023), or seeking different models of patient-practitioner interaction (Van Sickle et al., 2003; Zhang et al., 2023).

#### 3.4.2 Practice and challenges of shared decision-making (SDM)

Shared Decision-Making (SDM) is widely recognized for its potential to enhance families’ understanding of treatment, satisfaction, and adherence, ultimately leading to improved health outcomes (Gandhi et al., 2013; Rivera-Spoljaric et al., 2014; Gijsen et al., 2024; Zhou et al., 2025; Yi et al., 2024; Fan et al., 2023). However, effectively integrating it into routine clinical practice faces numerous challenges. First are communication barriers: significant obstacles may exist between healthcare providers and families regarding symmetry in information understanding, effective communication of treatment expectations, and the establishment of mutual trust (Sleath et al., 2011; Anagnostou et al., 2025a). Language and cultural differences (Santos Malavé et al., 2019; Reeves et al., 2020; Gijsen et al., 2024; Sleath et al., 2011), gaps in family health literacy levels, and limited consultation time in clinical practice (Rivera-Spoljaric et al., 2014; Yi et al., 2024) all constitute common communication barriers. Furthermore, effective communication strategies for pediatricians to promote treatment adherence are still under exploration (Gijsen et al., 2024; Fan et al., 2023).

Second is the complexity of SDM implementation. Successful implementation of SDM requires healthcare professionals to possess adequate communication skills and effective support tools, but relevant professional training and mature decision aids are currently relatively scarce. Research indicates that even under ideal conditions, the extent of SDM implementation may be insufficient; for example, primary care physicians might focus more on managing acute symptoms while neglecting the SDM aspects of chronic disease management. Meanwhile, the children themselves are often not actively included in the decision-making process (Sleath et al., 2011; Anagnostou et al., 2025a).

Third is the heterogeneity of family participation. Families differ in their willingness and ability to participate in SDM, which can be influenced by various factors such as their cultural background, educational level, self-confidence, and level of trust in the healthcare system (Yi et al., 2024; Anagnostou et al., 2025b). A deep understanding of the specific barriers to SDM from the parents’ perspective [e.g., difficulties encountered when making decisions about exercise prescriptions (Fan et al., 2023; Anagnostou et al., 2024)] is crucial for designing effective interventions. Particularly when facing serious conditions (such as developing a PICU discharge plan), parents’ decision-making processes can be fraught with difficulties and challenges (Anagnostou et al., 2025a; Moloney et al., 2023).

#### 3.4.3 Facilitators and tools for SDM

To promote the application of SDM in clinical practice, the following strategies can be adopted: (1) Improving Physician-Patient Communication: The core lies in building a trusting physician-patient relationship (Doerr, 2001; Pongdee and Li, 2025), using clear and empathetic communication methods, and proactively exploring and responding to families’ values, preferences, and concerns (Anderson et al., 2023; Anagnostou et al., 2025b; Cornelius et al., 2024; Fiks et al., 2015). (2) Decision Support Tools: decision aids, through structured information presentation, can help families better understand the pros and cons of different treatment options, weigh potential benefits against risks, and thus promote informed decision-making. Initial attempts have been made to develop decision aids for food allergy oral immunotherapy (OIT) (Anagnostou et al., 2024; Moloney et al., 2023; Ross et al., 2022; McWilliams et al., 2018), and similar tools also hold potential for application in the field of childhood asthma management (Fiks et al., 2015; Masrour et al., 2024). (3) Utilizing Information Technology: Technological means such as clinical decision support tools integrated into Electronic Health Records (EHR) (Fiks et al., 2015; Masrour et al., 2024), patient portals (Ross et al., 2022; McWilliams et al., 2018; Skeen et al., 2025; Bryant-Stephens et al., 2024), and mobile health applications (Masrour et al., 2024; Montalbano et al., 2020) are considered promising methods for supporting SDM, facilitating information sharing, and enhancing physician-patient communication (Sweenie et al., 2024; Kranjac et al., 2025). However, when applying these technologies, attention must be paid to user-friendly design, and vigilance is needed regarding the potential to exacerbate the digital divide (Masrour et al., 2024; Montalbano et al., 2020). Emerging algorithmic decision-making support systems (ADMSs) are also being explored, but their goal-setting needs to fully integrate the perspectives of children, parents, and clinicians (Skeen et al., 2025; Sweenie et al., 2024). (4) Integrating Multifaceted Factors: Effective SDM needs to go beyond mere medication choices and integrate non-pharmacological management strategies such as environmental control, social support, and behavioral interventions into decision-making considerations (Bryant-Stephens et al., 2024; Kranjac et al., 2025).

In summary, in the management of severe childhood asthma, SDM is a key pathway to achieving individualized, family-centered care. Despite facing multiple barriers including communication, time constraints, lack of tools, and varying family participation capabilities, it is hoped that by improving physician-patient communication, developing and applying decision support tools, and utilizing information technology, the effective implementation of SDM can be promoted, thereby better meeting family needs and optimizing treatment decisions.

## 4 Discussion

This review focuses on the complex considerations families face when choosing between omalizumab and CAM for the treatment of severe childhood asthma. Through a systematic review of existing literature and targeted supplementary searches, it aims to provide a deeper and more scholarly analysis. The discussion section will revolve around four core arguments, integrating the research findings and exploring their implications for clinical practice and future research.

### 4.1 Prioritizing family needs: the shift from clinical indicators to quality of life and patient-reported outcomes

The research findings strongly confirm that when families evaluate treatment options, their concerns extend far beyond traditional clinical efficacy indicators. Improving and maintaining an acceptable quality of life (QoL) and positive patient-reported outcomes (PROs) are core expectations and treatment goals for families (Gandhi et al., 2013; Zhou et al., 2025; Hossny et al., 2017; Aldirawi et al., 2025; Kasse et al., 2024; Williams et al., 2023; Zhou et al., 2025; Aldirawi et al., 2025; Kasse et al., 2024; Khaleva et al., 2025; Williams et al., 2023; Ip et al., 2018). This emphasis is critical, as factors like QoL are not only paramount to families but are also linked to broader aspects of child wellbeing, including behavioral issues (Montalbano et al., 2020; Castro et al., 2015). This implies that families place greater importance on whether treatment allows the child to return to a normal life trajectory, reduces the disease’s limitations on daily activities, and alleviates the operational burden on the entire family. Concerns about side effects, particularly regarding the long-term safety of medications [including “steroid phobia” (Zhao et al., 2023; Bingemann et al., 2024) and worries about unknown risks of biologics (Cornelius et al., 2024; Heidrich et al., 2017)], along with the expectation that CAM might offer a more “natural” or lower-side-effect alternative (Berg et al., 2016; Courtney et al., 2005; Courtney et al., 2005; Adams et al., 2007), profoundly influence family preferences. Therefore, clinical decisions should not be based solely on objective indicators but should place QoL and PROs in positions of equal importance, incorporating family priorities (Michalopoulou et al., 2016; Van Sickle et al., 2003) into the co-creation of treatment goals.

### 4.2 Subjectivity of risk perception and the evidence vacuum: understanding “explanatory models” and communication challenges

This review reaffirms the significant evidence gap regarding research into families’ specific risk perceptions of omalizumab and CAM. The literature search failed to identify high-quality empirical studies directly and deeply exploring this topic. This leaves our understanding of family risk perception largely reliant on theoretical inference and analysis of related phenomena [such as motivations for CAM use (Courtney et al., 2005; Adams et al., 2007), reasons for treatment non-adherence (Zhao et al., 2023; Bingemann et al., 2024)]. Families’ risk assessment is not purely based on objective data but is constructed through their subjective “Explanatory Models” (EMs) (Zhao et al., 2023; Bingemann et al., 2024) and treatment beliefs [such as “natural equals safe” (Courtney et al., 2005; Adams et al., 2007) or “steroid phobia” (Jones et al., 2018; Golding et al., 2021)]. Risk perception regarding omalizumab may be amplified by “dread risks” and “uncertainty” (Cornelius et al., 2024; Heidrich et al., 2017), while the risks of CAM are prone to underestimation (Gandhi et al., 2013; Zhou et al., 2025). This cognitive bias poses a serious challenge to clinical communication: physicians need not only to convey risk information but also to explore and understand families’ unique EMs and belief systems to bridge the cognitive gap and achieve effective risk communication.

### 4.3 The hidden costs of treatment burden: from theoretical recognition to practical assessment

The importance of treatment burden as a key “hidden cost” impacting family QoL, decision-making, and adherence is well-supported by the literature. Severe asthma management not only brings the burden of the disease itself (Williams et al., 2023; Valero-Moreno et al., 2018; Ip et al., 2018; Graves et al., 2007) but also superimposes a multidimensional treatment burden arising from executing complex treatment regimens, encompassing time, energy, financial costs, and significant psychosocial stress (Bingemann et al., 2024; Golding et al., 2021; Graves et al., 2007; Valero-Moreno et al., 2018; Adams et al., 2004; Ziaian et al., 2006; Khalsi et al., 2024; Amirav et al., 2020). The trade-offs families make between disease burden and treatment burden directly influence their “volitional non-adherence” behaviors (Ischander and Lozowski-Sullivan, 2022; Adams et al., 2004; Ziaian et al., 2006; Valero-Moreno et al., 2018; Prather et al., 2020; Ramsey et al., 2023). Although treatment burden theory (Ischander and Lozowski-Sullivan, 2022; Valero-Moreno et al., 2018) has been proposed and its impact on QoL confirmed (Ziaian et al., 2006; Prather et al., 2020), systematic assessment and management of treatment burden remain widely lacking in clinical practice. Integrating the assessment of treatment burden into routine clinical workflows and making it a core topic in SDM discussions is crucial for alleviating family stress, improving adherence, and enhancing outcomes.

### 4.4 Shared decision-making (SDM) and health equity: intertwined challenges

SDM, as an ideal decision-making model, is widely recognized for its value in childhood asthma management (Rivera-Spoljaric et al., 2014; Gijsen et al., 2024; Yi et al., 2024; Fan et al., 2023), especially in complex decisions requiring trade-offs [such as choosing biologics (Cornelius et al., 2024; Heidrich et al., 2017) or OIT (Anagnostou et al., 2025b; Anagnostou et al., 2024; Moloney et al., 2023; Fiks et al., 2015; Ross et al., 2022; McWilliams et al., 2018)]. However, the effective implementation of SDM faces numerous practical barriers, including communication difficulties, time constraints, lack of tools, and variations in families’ ability and willingness to participate (Sleath et al., 2011; Yi et al., 2024; Fan et al., 2023; Anagnostou et al., 2025a; Anagnostou et al., 2025a; Anagnostou et al., 2025b; Anagnostou et al., 2024; Moloney et al., 2023). Notably, health equity issues are closely related to the practice of SDM. Substantial evidence indicates that factors such as socioeconomic status, race/ethnicity, and cultural background significantly influence childhood asthma outcomes (Sweenie et al., 2024; Kranjac et al., 2025; Lo et al., 2024; Rodríguez et al., 2024; Lo et al., 2024; Rodríguez et al., 2024; Fiks et al., 2014; Ludden et al., 2022) and may limit the ability of vulnerable families to effectively participate in SDM (Santos Malavé et al., 2019; Reeves et al., 2020; Gijsen et al., 2024; Sleath et al., 2011). Therefore, promoting SDM requires not only technical improvements [such as developing decision aids (Anagnostou et al., 2024; Moloney et al., 2023; Fiks et al., 2015; Ross et al., 2022; McWilliams et al., 2018; Masrour et al., 2024) or utilizing information technology (Ross et al., 2022; McWilliams et al., 2018; Masrour et al., 2024; Skeen et al., 2025; Fiks et al., 2014; Skeen et al., 2025; Bryant-Stephens et al., 2024; Montalbano et al., 2020; Sweenie et al., 2024; Agache et al., 2020; Castro et al., 2015)] but also addressing health inequalities at a systemic level. This includes, for example, implementing community health worker programs (Sweenie et al., 2024; Lo et al., 2024) or providing culturally adapted interventions (Ludden et al., 2022; Giovannini et al., 2019) to ensure all families can equally access information, express preferences, and participate in decision-making.

### 4.5 Limitations

This review strives to enhance its academic rigor and evidence base, yet limitations remain. The narrative review methodology may introduce selection bias. Furthermore, the discussion in this review primarily centers on omalizumab, failing to provide an equally in-depth analysis and comparison of other biologic agents already in clinical use for asthma, such as mepolizumab, reslizumab, benralizumab, and dupilumab. We selected omalizumab as the main subject of investigation considering that, as the first biologic agent used for asthma treatment, it has been in clinical application for over 10 years. Its long-term efficacy, safety, and data in diverse populations are more comprehensive and mature, providing a solid foundation for relevant discussions (Giovannini et al., 2019; Agache et al., 2020; Castro et al., 2015). However, this focus also means that the review does not fully represent the application and considerations of other biologic agents in specific populations or particular asthma phenotypes, which constitutes a significant limitation of this study. The literature predominantly reflects the perspectives of parents/caregivers, with an insufficient representation of children’s viewpoints. Differences in the geographical and cultural backgrounds of the studies may affect the generalizability of the results. The definition of CAM is broad and fails to elaborate on considerations for different types of CAM. Most critically, direct and in-depth research on families’ risk perception of specific treatments (especially omalizumab and various types of CAM) remains a major research gap.

### 4.6 Implications for clinical practice and future research directions

Based on the analysis results of this review, the following recommendations are proposed for clinical practice and future research.

#### 4.6.1 Implications for clinical practice

Based on the analysis results of this review, the following recommendations are proposed for clinical practice: First, clinical decision-making should place the improvement of quality of life (QoL) and patient-reported outcomes (PROs) among core treatment goals, and routinely use validated, standardized tools for assessment and monitoring. Second, healthcare professionals need to proactively inquire about and understand families’ explanatory models (EMs) and core beliefs regarding disease etiology, course, and treatment efficacy. They should effectively identify and appropriately address negative perceptions that may interfere with treatment decisions and adherence, such as “steroid phobia”. Third, treatment burden, encompassing time, energy, financial, and psychosocial costs, should be incorporated into routine clinical assessment systems to identify families experiencing high burden and connect them with necessary support resources. Concurrently, risk communication strategies must be optimized, employing more effective and empathetic approaches to explain treatment-related risks and uncertainties, focusing on bridging the gap between objective risk data and families’ subjective perceptions. Finally, Shared Decision-Making (SDM) should be actively promoted, utilizing existing decision aids and paying special attention to the needs of families from diverse socioeconomic backgrounds and cultural groups. Ensure the provision of culturally sensitive and easily understandable information support to promote equitable decision-making.

#### 4.6.2 Future research directions

To further deepen the understanding of family decision-making mechanisms in severe childhood asthma and optimize management strategies, future research should focus on the following key areas: First, there is an urgent need for high-quality qualitative and quantitative research to deeply explore the specific perceptions, concerns, and underlying formation mechanisms among family members (including the children themselves) regarding the long-term safety of omalizumab and the potential risks of various Complementary and Alternative Medicine (CAM) therapies. This is crucial to fill the significant evidence gap in current risk perception. Second, longitudinal studies should be conducted to track and evaluate the dynamic changes in treatment burden derived from different treatment pathways (including biologics and CAM) over time, and their actual impact on treatment adherence and long-term clinical outcomes. Third, efforts are needed to design and rigorously evaluate Shared Decision-Making (SDM) interventions and decision aids tailored to the characteristics of childhood asthma management. Particular attention should be paid to integrating risk communication and treatment burden assessment functions, and their effectiveness and applicability should be tested across populations with diverse cultural backgrounds and health literacy levels. Furthermore, it is necessary to systematically evaluate the effectiveness and cost-effectiveness of intervention programs aimed at reducing health inequalities in childhood asthma management (such as community health worker programs or culturally adapted interventions). Finally, for specific CAM therapies that already have preliminary evidence or are widely used clinically, more rigorously designed randomized controlled trials (RCTs) with larger sample sizes should be conducted to scientifically and definitively assess their actual efficacy and safety in childhood asthma management.

## 5 Conclusion

Management decisions for severe childhood asthma represent a complex process interwoven with clinical evidence, family values, subjective experiences, and social contexts. By integrating literature evidence, this review emphasizes the need for clinical practice to shift from a purely biomedical model to a more family-centered approach. This implies moving beyond symptom control to focus on QoL and PROs; understanding and responding to families’ risk perceptions formed based on their unique explanatory models and beliefs; identifying and striving to alleviate the multidimensional burdens imposed by treatment; and actively promoting SDM while concurrently addressing health inequalities. Although biologics like omalizumab offer new therapeutic hope, and CAM meets the specific needs of some families, the choice is not black and white. Future research should focus on filling critical evidence gaps, particularly regarding in-depth studies on risk perception, and develop effective tools and strategies to support clinical practice. The ultimate goal is to empower families, optimize individualized treatment decisions, and improve the long-term health and wellbeing of all affected children and their families.

## Data Availability

The original contributions presented in the study are included in the article/supplementary material, further inquiries can be directed to the corresponding author.
